# Roles of LRRK2 and its orthologs in protecting against neurodegeneration and neurodevelopmental defects

**DOI:** 10.3389/fcell.2025.1569733

**Published:** 2025-04-30

**Authors:** An Phu Tran Nguyen, Linh Thi Nhat Nguyen, Bailey A. Stokke, Christopher C. Quinn

**Affiliations:** Department of Biological Sciences, University of Wisconsin-Milwaukee, Milwaukee, WI, United States

**Keywords:** LRRK2, Parkinson’s disease, autism, intellectual disability, neurodegeneration

## Abstract

In humans, variants in the *LRRK2* gene are the most prevalent risk factors for Parkinson’s disease (PD). Whereas studies in model organisms have long indicated that the orthologs of the wild-type LRRK proteins protect against neurodegeneration, newer findings indicate that they also protect against neurodevelopmental defects. This normal role of the LRRK proteins can be disrupted by either gain-of-function (GOF) or loss-of-function (LOF) mutations, leading to neurodegeneration and neurodevelopmental defects. Here, we review the roles of the LRRK proteins and their orthologs in these processes, with a focus on autophagy as a common factor that may mediate both of these roles. We also highlight the potential for experiments in vertebrate and invertebrate model systems to synergistically inform our understanding of the role of LRRK proteins in protecting against neurological disorders.

## Introduction

Variants in the *LRRK2* gene have been associated with Parkinson’s disease (PD) in humans and studies of model organisms suggest that orthologs of this gene protect against both age-related neurodegeneration and defects in neurodevelopment. For example, in mice, neurodegeneration can be caused by either a gain-of-function variant in *LRRK2* or by a double mutation that deletes both *LRRK2* and its functional homolog *LRRK1* (Dusonchet et al., 2011; Ramonet et al., 2011; Kang et al., 2024). More recently, it has become apparent that *LRRK2* and its orthologs also protect against neurodevelopmental defects. For example, gain-of-function and loss-of-function mutations in *LRRK2* cause axon guidance defects in mice (Onishi et al., 2020). Likewise, loss of function mutations in the *lrk-1* ortholog of the *LRRK* genes also causes axon guidance defects in *Caenorhabditis elegans* (Kuwahara et al., 2016; Drozd et al., 2024). These observations suggest that the normal role of the LRRK proteins (Human LRRK1, Human LRRK2, *C. elegans* LRK-1, and *Drosophila* dLRRK) is to protect against both neurodegeneration and defects in neurodevelopment. Moreover, these normal roles of the LRRK proteins can be disrupted by either gain-of-function or loss-of-function mutations. Here, we review the roles of the LRRK proteins in protecting against neurodegeneration and neurodevelopmental defects and consider the regulation of autophagy as a common factor for both of these functions.

### Overview of the LRRK2 and LRRK1 proteins

**FIGURE 1:**
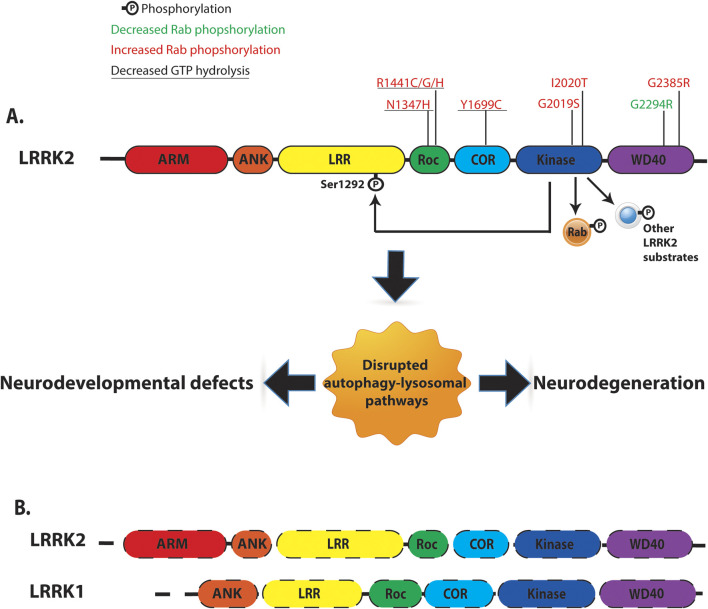
*Overview of human LRRK2/LRRK1 structure and function.* **(A)** LRRK2 is a multi-domain protein containing Armadillo domain (ARM, Red), Ankyrin repeat (ANK, Orange), Leucine rich repeat (LRR, Yellow), Ras-of-complex (Roc, Green), C-terminal of ROC (COR, Blue), Kinase (KIN, Navy), and WD40 (Purple). LRRK2 contains two key enzymatic domains: a Roc-GTPase domain and a kinase domain. PD-linked LRRK2 variants with reported kinase and GTPase activities were indicated. LRRK2 kinase can phosphorylate LRRK2 itself at Ser1292 residue (auto-phosphorylation). Several intracellular substrates have been identified for LRRK2 kinase, including a subset of Rab-GTPases. **(B)** Comparison of domain organization of LRRK1 and LRRK2. LRRK1 and LRRK2 contain similar domain organization: Ankyrin repeat, Leucine rich repeat (LRR), Ras-of-complex, C-terminal of ROC, Kinase and WD40 domain. Dash lines represent loss of LRRK1 and LRRK2 expression.

LRRK2 is a large (286 kDa), multidomain, homodimeric protein that is ubiquitously expressed, with the highest levels detected in the kidneys, lungs, and brain. As a member of the Roco protein family, LRRK2’s structure includes several functional domains (Figure 1A): armadillo (ARM) repeats, ankyrin (ANK) repeats, leucine-rich repeats (LRR), a GTP-binding Ras of complex (ROC) domain coupled to C-terminal of ROC (COR), a catalytic kinase (KIN) domain, a WD40 domain, and an extended C-terminal αC-helix (Myasnikov et al., 2021). Notably, LRRK2 exhibits two enzymatic activities: a Ras-like GTPase and a kinase, a unique feature of certain Roco family proteins (Alessi and Pfeffer, 2024).

Within the Roco protein family, LRRK1 is a functional homolog of LRRK2, sharing similar LRR, ROC, COR, and kinase domains (Figure 1B) (Marin, 2008). Despite structural similarities, LRRK1 exhibits distinct mechanisms of autoinhibition/activation and physiological functions compared to LRRK2 (Metcalfe et al., 2023; Reimer et al., 2023). Autosomal recessive variants in the *LRRK1* gene that cause frameshift or truncating mutations in the C-terminal domain of the LRRK1 protein, likely lead to loss of function and are associated with osteosclerotic metaphyseal dysplasia, a severe metabolic bone disorder (Alessi and Pfeffer, 2024). Functionally, LRRK1 efficiently phosphorylates Rab7A at Ser72 but does not target Rab8A or Rab10, the primary LRRK2 substrates in cells (Malik et al., 2021).

### Pathogenic variants in the LRRK2 protein can cause Parkinson’s disease in humans

Mutations in the *LRRK2* gene are the most common genetic cause of familial autosomal dominant Parkinson’s disease (PD), accounting for 2%–40% of cases depending on the population studied (Mata et al., 2023). Clinically, the progression of symptoms and neuropathology in patients with LRRK2-associated PD (LRRK2-PD) are indistinguishable from those observed in sporadic PD cases (Aasly et al., 2005; Healy et al., 2008). Thus, investigations of LRRK2 are thought to be a platform for understanding the molecular mechanisms that underlie all forms of Parkinson’s.

Seven pathogenic missense mutations have been identified in LRRK2 (Figure 1A), located in the ROC-GTPase domain (N1347H, R1441 C/G/H), COR domain (Y1699C), and kinase domain (G2019S, I2020T). These mutations highlight the critical role of enzymatic activity in LRRK2 function. Mutations in the kinase domain (G2019S and I2020T) enhance LRRK2 kinase activity *in vitro*, while those in the ROC-COR domain (R1441 C/G/H and Y1699C) disrupt dimer stability and reduce GTPase activity (Nguyen and Moore, 2017). LRRK2 kinase phosphorylates various substrates, including a group of ∼14 Rab-GTPases (LRRK2-Rabs), implicating LRRK2 in endosomal and vesicle trafficking pathways (Steger et al., 2016). All seven pathogenic mutations increase LRRK2-Rab phosphorylation, suggesting a gain-of-function mechanism through enhanced kinase activity (Steger et al., 2016).

### LRRK2 GOF proteins cause age-related neurodegeneration in model organisms

Pathogenic LRRK2 missense mutations that cause increased kinase activity consistently cause axonal degeneration and neuronal cell death across various model systems. In *Drosophila*, expression of the common pathogenic LRRK2 mutant protein G2019S causes severe retinal degeneration, selective dopaminergic neuron loss, reduced climbing ability, and early mortality (Liu et al., 2008; Lin et al., 2010). Additionally, G2019S LRRK2 expression exacerbates tau-induced dendritic degeneration, microtubule fragmentation, and inclusion formation in fly neurons (Lin et al., 2010). In *C. elegans*, dopaminergic neuron-specific expression of pathogenic LRRK2 mutant proteins R1441C and G2019S induces age-dependent locomotor impairments, axonal degeneration, and dopaminergic neuronal loss (Yao et al., 2010; Cooper et al., 2015; Senchuk et al., 2021).

In mammalian models, overexpression of G2019S LRRK2 in mice using the PDGFβ promoter leads to progressive loss of dopaminergic neurons in the substantia nigra pars compacta (SNpc) by 19–20 months of age (Ramonet et al., 2011). Similarly, in rats, overexpression of G2019S LRRK2 via recombinant human adenoviral vectors (Ad5) in the nigrostriatal pathway results in progressive dopaminergic neuron loss in the SNpc (Dusonchet et al., 2011). Remarkably, neurodegenerative phenotypes associated with G2019S LRRK2 are kinase-dependent, as shown by the suppression of these phenotypes through expression of the kinase-dead mutant G2019S/K1906M or treatment with LRRK2 kinase inhibitors (Nguyen et al., 2020). Common pathological features observed in transgenic and adenoviral LRRK2 animal models include axonal abnormalities such as hyperphosphorylated tau accumulation, fragmented axons with spheroids and dystrophic neurites, increased Gallyas silver deposits, and APP-positive inclusions (Li et al., 2009; Li et al., 2010; Melrose et al., 2010; Dusonchet et al., 2011; Tsika et al., 2015; Yue et al., 2015; Nguyen et al., 2020).

### LOF mutations in *LRRK* genes cause age-related neurodegeneration in model organsisms

While gain-of-function mutations in LRRK2 proteins can cause axonal degeneration and neuronal death, evidence suggests that loss of LRRK proteins can also result in similar pathologies. *LRRK* loss-of-function mutations in *Drosophila* exhibit severe locomotor deficits, reduced tyrosine hydroxylase immunoreactivity, and atrophic dopaminergic neurons (Lee et al., 2007). In mice, deletion of the *LRRK2* gene alone does not cause brain phenotypes (Tong et al., 2010; Herzig et al., 2011). The lack of a pronounced brain phenotype in *LRRK2* knockout mice may be due to compensatory effects by LRRK1. Supporting this, deletion of both *LRRK1* and *LRRK2* leads to age-dependent, progressive loss of dopaminergic neurons in the SNpc and dopaminergic terminals in the striatum starting at 14 months of age (Giaime et al., 2017; Huang et al., 2022). Recently, Kang and colleagues demonstrated that specific deletion of both *LRRK1* and *LRRK2* in mouse dopaminergic neurons causes age-dependent progressive loss of SNpc dopaminergic neurons at 20–24 months of age (Kang et al., 2024). These findings underscore the critical roles of both LRRK1 and LRRK2 in maintaining dopaminergic neuron homeostasis in animal models.

Whereas LRRK family loss-of-function can cause neurodegeneration in animal models, the role of LRRK2 loss-of-function in humans remains uncertain. On one hand, analysis of predicted loss-of-function variants in the *LRRK1* and *LRRK2* genes failed to find any association with Parkinson’s disease (Blauwendraat et al., 2018). On the other hand, two *LRRK2* risk variants have been reported that may have loss-of-function effects. For example, LRRK2 G2385R is one of the most prevalent risk variants worldwide and is reported to cause a reduction in LRRK2 kinase activity *in vitro* and a reduction in LRRK2 stability in cells (Rudenko et al., 2012). However, some studies have reported that the LRRK2 G2385R variant increases LRRK2-Rab phosphorylation (Steger et al., 2016; Zhang et al., 2019; Kalogeropulou et al., 2022). Recently, another *LRRK2* loss-of-function variant, G2294R, has been identified in a patient with familial PD. Consistent with a loss-of-function mechanism, this variant reduces LRRK2 protein levels and LRRK2-mediated Rab10 phosphorylation in cells (Ogata et al., 2021). Together, these observations suggest the hypothesis that *LRRK2* loss-of-function variants can contribute to PD in humans. Nonetheless, more research will be needed to determine if and how LRRK2 loss of function contributes to PD.

### The LRRK proteins protect against defects in neurodevelopment in model organisms

In mice, wild-type LRRK proteins protect against defects in axon guidance, and this process is disrupted by either gain-of-function or loss-of-function alleles in the *LRRK* genes (Onishi et al., 2020). For example, knockout of either *LRRK1* or *LRRK2* causes axon guidance defects in the commissural axons of the spinal cord. Likewise, the double knockout of *LRRK1* and *LRRK2* causes axon guidance defects in the midbrain dopamine neurons. The LRRK2 G2019S gain-of-function mutant protein causes axon guidance defects in both spinal cord commissural neurons and mid brain dopamine neurons. These observations indicate that neurodevelopment can be disrupted by either GOF and LOF alleles of *LRRK2*, suggesting that precise regulation of LRRK2 activity is required for normal development.

Recent work has begun to reveal the mechanisms through which the LRRK proteins promote axon guidance. For example, LRRK proteins promote axon guidance by phosphorylating Frizzled3, thereby promoting its interaction with the planer cell polarity pathway. Moreover, observations of cultured neurons suggest that LRRK2 and the planer cell polarity pathway promote axon guidance by regulating the interaction between growth cones. Together, these observations suggest that LRRK2 promotes axon guidance by regulating the planer cell polarity protein, thereby influencing the interactions between growth cones.

Additional mechanistic insight for the role of the LRRK proteins in neuronal development comes from studies of the *C. elegans* LRK-1 ortholog of the LRRK1 and LRRK2 proteins. First, LRK-1 is required for termination of the growth of the PLM and ALM axons. These axons normally extend along the body wall and terminate at defined locations. Loss of LRK-1 function causes these axons to overshoot their normal termination sites (Kuwahara et al., 2016; Drozd et al., 2024). Second, LRK-1 is required for the polarized distribution of synaptic vesicle proteins within neurons. For example, the SNB-1 synaptic vesicle protein is normally localized to axons and excluded from dendrites. Loss of LRK-1 function causes SNB-1 to be localized in both axons and dendrites, suggesting that LRK-1 helps to exclude synaptic vesicle localization in dendrites (Sakaguchi-Nakashima et al., 2007). Moreover, LRK-1 can function with the UNC-16 (JIP3) adaptor protein and the SYD-2 active zone protein to regulate the protein composition and trafficking of synaptic vesicles precursors (Choudhary et al., 2017; Nadiminti et al., 2024).

In humans, defects in neurodevelopment are associated with neurodevelopmental disorders such as autism (ASD) and intellectual disability (ID). In this regard, it is interesting to note that growing evidence suggests a potential association between Parkinson’s disease and ASD/ID. For example, a small study has reported a high incidence of Parkinson’s disease in autistic individuals (Starkstein et al., 2015). Moreover, although unpublished, a recent large study has suggested that diagnosis of ASD and/or ID is a risk factor for Parkinson’s disease (Naddaf, 2024). Although this association is still not well understood, it could reflect the dual roles of LRRK proteins in protecting against both neurodegeneration and neurodevelopment.

### Regulation of autophagy may underlie the role of LRRK proteins in PD and neurodevelopment

There is growing evidence suggesting that abnormal LRRK2 activity disturbs the autophagy/lysosomal pathways, including mitophagy, the process of specific elimination of mitochondria by autophagy (Erb and Moore, 2020; Singh and Ganley, 2021). In cultured neurons, expression of G2019S and R1441C/H LRRK2 decreased autophagic flux or autolysosome maturation, possibly through disruption of axonal autophagosome transport (Schapansky et al., 2018; Wallings et al., 2019; Boecker et al., 2021; Dou et al., 2023). In *C. elegans*, G2019S or R1441C LRRK2 expression causes accumulation of LC3-homolog lgg-1:RFP, suggesting a reduction of autophagy flux (Saha et al., 2014). In mice, expression of G2019S or R1441C LRRK2 display increased numbers of large intra-axonal autophagic vacuoles (Ramonet et al., 2011). Mechanistically, the increase of LRRK2 kinase activity was shown to enhance the recruitment of JIP4, a motor adaptor known to bind to LRRK2-phosphorylated Rab proteins, to the autophagosomal membrane. Increased JIP4 levels induce abnormal recruitment and activation of kinesin-1, resulting in an unproductive tug-of-war between anterograde and retrograde motors bound to autophagosomes (Boecker and Holzbaur, 2021).

In contrast to the LRRK2 GOF variants, deletion of the LRRK2 gene caused an increase in autophagic flux in neurons cultured from postnatal day 1 rats, although this did not reach statistical significance (Wallings et al., 2019). Nonetheless, this LRRK2 deletion did cause a statistically significant increase in lysosomal protein degradation. The opposite effect was observed in the brains of ageing mice, where deletion of both LRRK2 and LRRK1 leads to anaccelerated decline of autophagic clearance and accumulation of large autophagic vacuoles in surviving dopaminergic neurons (Giaime et al., 2017; Huang et al., 2022). Taken together, these observations suggest that the deletion of the LRRK genes might have opposite effects on autophagy in young and old neurons. Consistent with this idea, loss of LRRK2 enhances autophagy in young rat kidneys and decreases autophagy in old rat kidneys (Tong et al., 2012).

Work in multiple systems has implicated LRRK2 mutations in the dysregulation of mitophagy, a selective form of autophagy that is critical for the homeostasis of mitochondria. Studies of fibroblasts and neurons derived from patients carrying the G2019S or R1441C LRRK2 mutations revealed abnormalities in mitochondrial morphology, and an increase of mitochondrial DNA damage (Mortiboys et al., 2010; Sanders et al., 2014; Wauters et al., 2020). In *C.elegans*, G2019S or R1441C LRRK2 expression increased the response of the mitochondrial hsp6 reporter to stress (Saha et al., 2014). In mice, G2019S LRRK2 expression was shown to induce progressive mitochondrial morphology changes and reduce basal mitophagy as indicated by the reduction of fluorescent reporter for mitophagy (“mito-QC”) (Yue et al., 2015; Singh et al., 2021). Mechanistically, LRRK2 was shown to form a complex with Miro, which is required for its efficient removal during PINK1/Parkin-dependent mitophagy (Hsieh et al., 2016). Expression of LRRK2 G2019S disrupted Parkin-dependent mitophagy, potentially via reducing Parkin’s interaction with outer mitochondrial membrane proteins, including the fission regulating GTPase DRP-1 (Bonello et al., 2019). Additionally, LRRK2 mutations impair depolarization-induced mitophagy through inhibition of mitochondrial accumulation of Rab10, a downstream substrate of LRRK2 (Wauters et al., 2020).

Emerging evidence suggests that the role of the LRRK proteins in axon development is also mediated through dysregulation of autophagy. This idea is supported by interactions between mutations in the genes that encode the UNC-16 (JIP3) adaptor protein, the LRK-1 ortholog of LRRK2, and the WDFY-3 selective autophagy protein (Drozd et al., 2024). UNC-16 is required for the retrograde transport of late endosomes and autophagosomes and its loss of function causes axonal accumulation of late endosomes and autophagosomes, which contain LRK-1 protein (Hill et al., 2019; Celestino et al., 2022; Drozd et al., 2024). Moreover, loss of *unc-16* causes overextension of the PLM axon and this phenotype can be suppressed by loss of *lrk-1* function (Drozd et al., 2024). The PLM axon overextension phenotype can also be suppressed by loss of *wdfy-3*, which encodes a selective autophagy protein. These observations suggest that excessive activity of LRK-1 and WDFY-3 might cause axon overgrowth in *unc-16* mutants. Furthermore, no additional suppression of this phenotype is observed in in *lrk-1*;*wdfy-3*;*unc-16* triple mutants, suggesting that *wdfy-3* and *unc-16* function in a genetic pathway with each other.

Based on these observations, we hypothesize that LRK-1 and WDFY-3 function within a pathway that can promote axon extension and that excessive accumulation of these proteins in the axon can cause axon termination defects. Moreover, it is interesting to note that that the *C. elegans* WDFY-3 protein is an ortholog of the human WDFY3 selective autophagy protein, which is encoded by a gene that has been associated with ASD and ID (Fu et al., 2022). Therefore, we hypothesize that the WDFY3 and LRRK proteins could function together to protect against autism.

Studies of cultured mammalian neurons also support the idea that the role of the LRRK family in axon growth is mediated through the dysregulation of autophagy. Multiple studies have indicated that the LRRK2 G2019S mutation reduces the growth of axons and dendrites in cultured primary neurons (Stafa et al., 2012; Sepulveda et al., 2013; Stafa et al., 2014; Kang et al., 2024). One study of the SH-SY5Y neuroblastoma cell line has also found that the LRRK2 G2019S mutation causes an accumulation of autophagosomes within neurites along with a decrease in neurite length (Plowey et al., 2008). Moreover, both of these phenotypes can be suppressed by knockdown of either the ATG7 or LC3 autophagy proteins. These observations suggest that LRRK2 G2019S disrupts axon growth through the dysregulation of autophagy. These observations are also consistent with the hypothesis that wildtype LRRK2 has a role in regulating axon growth through the regulation of autophagy.

## Discussion

Here, we have reviewed the roles of the LRRK proteins in protecting against neurodegeneration and promoting axon development in multiple model organisms. We have also considered evidence that the LRRK family regulates autophagy, and that disruption of autophagy is likely to underlie the neurodegenerative and neurodevelopmental phenotypes of *LRRK* gene variants. Moreover, we have discussed genetic interactions suggesting that the LRK-1 ortholog of LRRK2 regulates axon development by functioning in a pathway with the ortholog of the WDFY3 selective autophagy protein (aka Alfy), which is encoded by an autism-associated gene. Taken together, these observations suggest the hypothesis that the role of the LRRK proteins in regulating autophagy could underlie their roles in protecting against neurodegeneration and neurodevelopmental defects. We also hypothesize that these dual roles for LRRK proteins could explain the association between ASD and PD. Further investigation of this hypothesis will require additional work in model organisms and further human genetic analysis.

A key question for future investigation is the potential involvement of *LRRK2* in protecting against neurodevelopmental disorders. Given the role of *LRRK* genes in protecting against neurodevelopmental defects in mice, *Drosophila* and *C. elegans*, we propose that they might protect against neurodevelopmental disorders in humans. Thus far, investigations of *LRRK2* association with neurodevelopmental disorders have been inconclusive. On one hand, comparative genomic mapping with microdeletions has suggested that deletion of *LRRK2* can cause a syndrome that presents as intellectual disability and autism (Labonne et al., 2020). On the other hand, a large study of human *LRRK2* loss of function variants failed to identify an association with any disorders (Whiffin et al., 2020). One possible reason for this discrepancy is that autism may occur as a result of a genetic interaction between *LRRK2(LOF)* and variants in other neurodevelopmental genes. Thus, the microdeletions could cause autism by synergizing with variants in one or more other autism-associated genes. Therefore, we propose that an important goal for future research with model organisms will be to identify synergistic genetic interactions between mutations in *LRRK* genes and neurodevelopmental disorder-associated genes. With regards to human genetic analysis, it may be useful to investigate a potential association between *LRRK2(GOF)* variants and neurodevelopmental disorders.

Another key question for future investigation is the potential involvement of the *WDFY3* gene in protecting against Parkinson’s disease and other neurodegenerative disorders. Considering the genetic interactions between *wdfy-3* and *lrk-1* in *C. elegans*, we propose that the *WDFY3* gene could be involved in protecting against Parkinson’s disease. Although *WDFY3* gene has not been associated with Parkinson’s, the WDFY3 protein has been implicated in mitophagy, which is thought to be involved in Parkinson’s (Gao et al., 2017; Napoli et al., 2018). In addition, *WDFY3* has been implicated in protecting against Huntington’s disease, suggesting that it can protect against neurodegeneration (Fox et al., 2020). To further investigate the role of WDFY3 in neurodegeneration, future investigations may seek to explore genetic interactions between variants in *WDFY3* and *LRRK2* in animal models of Parkinson’s disease.

## Data Availability

The original contributions presented in the study are included in the article/supplementary material, further inquiries can be directed to the corresponding authors.
